# Finite element analysis of the influence of fragment size on biomechanical outcomes of various fixation techniques for posterolateral tibial plateau fractures

**DOI:** 10.3389/fbioe.2026.1676886

**Published:** 2026-01-28

**Authors:** Zhenghui Hu, Yanze Xia, Gaolong Shi, Chenying Wu, Liubing Li

**Affiliations:** Department of Orthopedics Surgery, The Second Affiliated Hospital of Soochow University, Soochow, Jiangsu Province, China

**Keywords:** finite element analysis, Fragment Size, L-shaped plate, posterolateral tibial plateau fracture, T-shaped plate

## Abstract

**Objective:**

This study aimed to compare the biomechanical stability of different-sized posterolateral tibial plateau fracture fragments under various internal fixation methods using three-dimensional finite element analysis (FEA) and to evaluate the influence of fragment size on displacement and stress distribution with L-shaped and posterior T-shaped plate fixation.

**Methods:**

Three-dimensional FEA models of posterolateral tibial plateau fractures were constructed based on computed tomography (CT) data of an adult tibia. Large and small fracture fragments were simulated, with fixation using an L-shaped plate (large fragment: dual-screw transverse arm fixation; small fragment: single-screw transverse arm fixation) or a posterior T-shaped tibial plate (large fragment: four-screw fixation; small fragment: three-screw fixation). Axial loads of 250 N, 500 N, and 750 N were applied to each model. Maximum displacement and maximum von Mises stress of the fixation constructs were recorded and compared under a 750 N load.

**Results:**

Under a 750 N axial load, FEA revealed: (1) L-shaped plate fixation group: the large-fragment model (dual-screw transverse arm) exhibited a maximum fixation displacement of 0.32 mm and a maximum von Mises stress of 349 MPa, whereas the small-fragment model (single-screw transverse arm) showed a maximum displacement of 0.37 mm and a maximum stress of 454 MPa. (2) Posterior T-shaped plate fixation group: the large-fragment model (four screws) demonstrated a maximum displacement of 0.29 mm and a maximum stress of 189 MPa, while the small-fragment model (three screws) had a maximum displacement of 0.32 mm and a maximum stress of 176 MPa. Overall displacement differences across groups were minimal, and the stress values of all fixation constructs remained below their yield strength, indicating no risk of failure.

**Conclusion:**

Finite element analysis indicates that fragment size significantly affects the biomechanical stability of internal fixation for posterolateral tibial plateau fractures. For smaller fragments, L-shaped plate fixation with a single-screw transverse arm results in significantly elevated fixation stress. In contrast, posterior T-shaped plate fixation maintains favorable stability and lower stress, even with fewer proximal screws. Thus, for smaller posterolateral tibial plateau fracture fragments, posterior T-shaped plate fixation may represent a biomechanically superior option.

## Introduction

Tibial plateau fractures are common intra-articular knee injuries, with posterolateral fractures posing unique challenges due to their deep anatomical location, difficult surgical exposure, and frequent association with meniscal and ligamentous injuries (Wang B. et al., 2024; Park et al., 2017). Inadequate anatomical reduction or unstable fixation of these fractures can lead to articular surface incongruity, knee instability, and post-traumatic osteoarthritis, significantly impairing knee function and patient quality of life (Kim et al., 2022; Parkkinen et al., 2014).

Open reduction and internal fixation (ORIF) remains the primary treatment for posterolateral tibial plateau fractures, with commonly used implants including lateral L-shaped plates and posterior T-shaped tibial plates (Liu et al., 2025; Gu et al., 2024; Sauhta and Makkar, 2025). These fixation methods provide sufficient biomechanical stability to promote fracture healing and facilitate early postoperative functional rehabilitation. Previous studies have primarily focused on comparing the efficacy of different surgical approaches or plate types for stabilizing posterolateral fracture fragments, with biomechanical analyses demonstrating superior mechanical strength for posterior T-shaped plates (Zhang C. et al., 2023; Zhang et al., 2020). Cho et al. evaluated the fixation of posterolateral fragments using a modified anterolateral approach combined with a “rim plate,” concluding that this technique effectively stabilizes the posterolateral fragment while maintaining the convenience of anterior access (Cho et al., 2017). Solomon et al. compared anterolateral and posterolateral approaches for unilateral posterolateral tibial plateau fractures, noting that the posterolateral approach offers advantages in fracture exposure and placement of buttress plates (Solomon et al., 2013). Ren et al. developed a novel plate design that enables fixation of posterolateral fragments via a conventional anterolateral approach, demonstrating biomechanical performance superior to traditional fixation methods (Ren et al., 2022). Our prior studies indicated that using two or more screws to secure posterolateral fragments achieves satisfactory mechanical strength (Hu et al., 2023a; Hu et al., 2023b).

However, in clinical practice, the size and morphology of posterolateral fracture fragments often exhibit considerable variability (Chang et al., 2009). When fragments are small or irregularly shaped, they may not accommodate the standard number of screws. For instance, the transverse arm of an L-shaped plate may only allow placement of a single screw, or a posterior T-shaped plate may require fewer screws due to fragment constraints. Whether such reduced-screw fixation configurations can still provide sufficient initial stability to withstand the physiological loads required for early functional rehabilitation remains poorly supported by biomechanical evidence. Therefore, evaluating the impact of fragment size on the biomechanical performance of different fixation methods, particularly when screw numbers are limited, is critical for guiding individualized clinical fixation strategies.

Finite element analysis (FEA), a well-established biomechanical research method, enables *in vitro* simulation of complex interactions between bone and internal fixation devices while precisely controlling variables to evaluate mechanical distributions under various conditions (Zhang B. B. et al., 2023; Wei et al., 2023; Yan et al., 2024). Accordingly, this study employed FEA to develop models of posterolateral tibial plateau fractures with varying fragment sizes, comparing the biomechanical stability of L-shaped plates and posterior T-shaped tibial plates when screw numbers are limited by fragment size. The aim was to provide a mechanical basis for selecting optimal internal fixation strategies tailored to the specific characteristics of fracture fragments in clinical practice.

## Materials and methods

**FIGURE 1:**
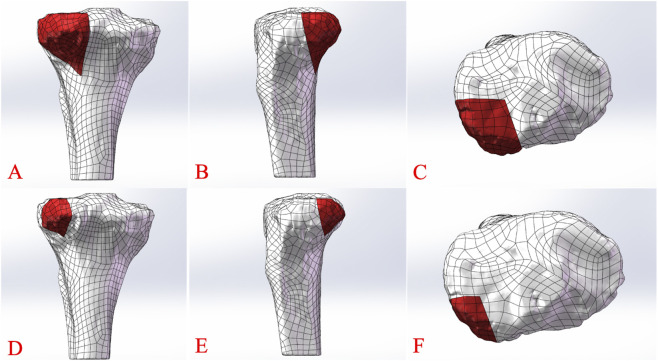
Schematic diagram of meshing of the finite element model of the posterolateral tibial plateau fracture. **(A–C)** Coronal, sagittal, and axial images of the large fracture fragment model. **(D–F)** Coronal, sagittal, and axial images of the small fracture fragment model.

This study employed FEA to evaluate the biomechanical stability of internal fixation methods for posterolateral tibial plateau fractures with varying fragment sizes, conducted from May to June 2025 at Shanghai Detection Technology Co., Ltd., Shanghai, China. Proximal tibial locking compression plates (3.5-mm LCP, Kanghui) and posterior T-shaped tibial plates (2.7-mm TP, Kanghui) were used as internal fixation devices. Software tools included Mimics 15.0 (Materialise, Belgium) for image processing, Geomagic 2017 (Geomagic, United States) for data scanning and 3D model conversion, Solidworks 2021 (Dassault Systèmes, United States) for three-dimensional modeling, and Ansys 13.0 (Ansys, Inc., United States) for FEA simulation. A healthy 30-year-old male volunteer (height: 170 cm, weight: 60 kg) was selected, and thin-layer computed tomography (CT) scans from the knee to the ankle were performed using a 64-slice CT scanner, with images stored in DICOM format. This study was approved by the institutional ethics committee (Approval No. JD-HG-2025–60). CT images were imported into Mimics 15.0, where threshold segmentation, region growing, and three-dimensional reconstruction techniques generated a detailed tibial model. Cancellous bone models were created using 3-matic 12.0, and corresponding solid models were developed in Geomagic 2017. These models were imported into Solidworks 2021 to construct four internal fixation configurations, meshed with tetrahedral elements (Solid187 mesh type). Fracture and fixation models were designed based on validated prior studies (Figures 1A–C) (Hu et al., 2023a; Hu et al., 2023b). To simulate smaller fragments, the fracture block was uniformly scaled down. Considering that posterolateral tibial plateau fractures fixed via the inverted L-shaped approach typically utilize posterior T-shaped plates, the coronal fracture model was adjusted to limit medial screw placement for T-shaped plates (Figure 1D). As prior studies indicate that posterolateral fragments are often wedge-shaped, the fracture line was extended posteriorly for L-shaped plate fixation via the lateral approach, allowing only single-screw transverse arm fixation (Figures 1E,F) (Markhardt et al., 2009; Vosoughi et al., 2025). In Solidworks 2021, 3.5-mm LCP and 2.7-mm TP models were imported and assembled with the fracture models. Four fracture models were established: Group A (large fragment, lateral LCP), Group B (large fragment, posterior TP), Group C (small fragment, lateral LCP), and Group D (small fragment, posterior TP). Finite element node and element counts are presented in Table 1. The four models were imported into Ansys 13.0 for simulation. Materials (tibial cortical bone, cancellous bone, plates, and screws) were assumed to be homogeneous, isotropic, linear elastic, with properties listed in Table 2. A friction coefficient of 0.15 was set between implants and fracture fragments. Considering a 60-kg adult male, knee joint loads during normal walking (2–3 times body weight) were approximated, with a 55:45 load distribution between medial and lateral tibial plateaus. Axial loads of 250 N, 500 N, and 750 N were applied parallel to the tibial plateau’s longitudinal axis to simulate posterolateral fragment loading. Compressive displacement, maximum von Mises stress, and stress distribution were analyzed under these load conditions.

**TABLE 1:** Node and element counts for the four models.

​	Number of elements	Number of unit nodes	Unit type	Unit size
Model A bone	129665	196509	C3D10	2
Fracture fragment	12376	19147	C3D10	2
Internal fixation	78748	130162	C3D10	1
Model B bone	129665	196509	C3D10	2
Fracture fragment	12376	19147	C3D10	2
Internal fixation	104907	158671	C3D10	1
Model C bone	141692	214319	C3D10	2
Fracture fragment	4157	6841	C3D10	2
Internal fixation	78748	130162	C3D10	1
Model D bone	141692	214319	C3D10	2
Fracture fragment	4157	6841	C3D10	2
Internal fixation	104907	158671	C3D10	1

**TABLE 2:** Material properties of the four groups of models.

Material	Elastic modulus (MPa)	Poisson’s ratio
Cortical bone	14000	0.3
Cancellous bone	700	0.3
Titanium alloy	110000	0.3

## Results

This study performed FEA to evaluate the biomechanical behavior of four posterolateral tibial plateau fracture models with different fixation configurations (Model A: L-shaped plate, large fragment, dual-screw fixation; Model B: T-shaped plate, large fragment, four-screw fixation; Model C: L-shaped plate, small fragment, single-screw fixation; Model D: T-shaped plate, small fragment, three-screw fixation) under axial loads of 250 N, 500 N, and 750 N. Overall displacement, implant displacement, tibial displacement, and corresponding von Mises stress distributions were recorded and analyzed. Detailed data are presented in Table 3 (implant models) and Table 4 (tibial models).

**TABLE 3:** Displacement and Maximum von Mises Stress of Four Implant Models Under Different Axial Loads.

Models	250N		500N		750N	
	Displacement (mm)	von Mises Stress(MPa)	Displacement (mm)	von Mises Stress(MPa)	Displacement (mm)	von mises Stress(MPa)
A	0.10	164	0.21	216	0.32	349
B	0.09	63	0.18	142	0.29	189
C	0.12	120	0.25	170	0.37	454
D	0.10	59	0.21	131	0.32	176

**TABLE 4:** Displacement and Maximum von Mises Stress of Four Tibial Models Under Different Axial Loads.

Models	250N		500N		750N	
	Displacement (mm)	von Mises Stress(MPa)	Displacement (mm)	von Mises Stress(MPa)	Displacement (mm)	von Mises Stress(MPa)
A	0.11	67	0.23	83	0.34	223
B	0.10	29	0.20	67	0.31	190
C	0.13	54	0.26	107	0.40	281
D	0.11	36	0.23	79	0.36	104

### Displacement distribution under different loading conditions

**FIGURE 2:**
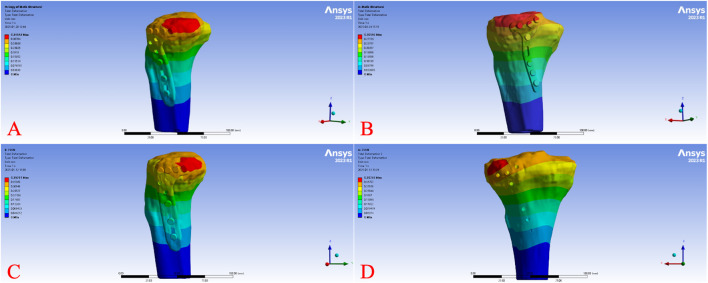
Overall displacement cloud diagram of different fixed models under 750 N axial load. Displacement distribution of the bone-implant construct under a 750 N axial load for Model **(A)** (L-shaped plate, large fragment, dual-screw fixation), Model **(B)** (T-shaped plate, large fragment, four-screw fixation), Model **(C)** (L-shaped plate, small fragment, single-screw fixation), and Model **(D)** (T-shaped plate, small fragment, three-screw fixation). Colors range from blue (minimal displacement) to red (maximum displacement).

**FIGURE 3:**
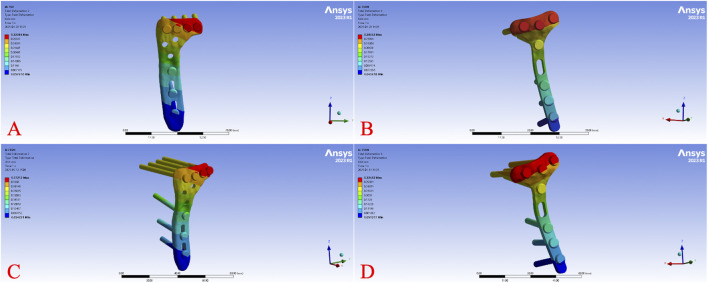
Internal fixation displacement cloud diagram of different fixation models under 750 N axial load. Displacement distribution of the implants (plates and screws) under a 750 N axial load for Model **(A)** (L-shaped plate, large fragment, dual-screw fixation), Model **(B)** (T-shaped plate, large fragment, four-screw fixation), Model **(C)** (L-shaped plate, small fragment, single-screw fixation), and Model **(D)** (T-shaped plate, small fragment, three-screw fixation).

**FIGURE 4:**
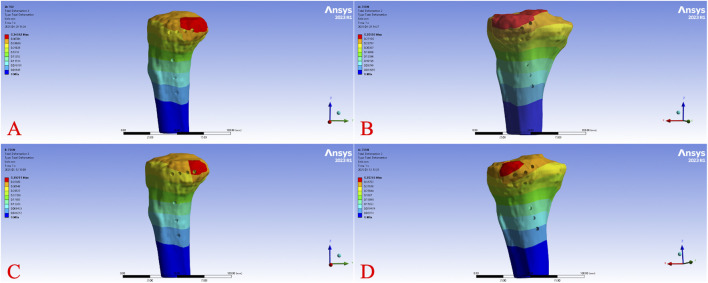
Tibial displacement cloud diagram of different fixation models under 750 N axial load. Displacement distribution of the tibia under a 750 N axial load for Model **(A)** (L-shaped plate, large fragment, dual-screw fixation), Model **(B)** (T-shaped plate, large fragment, four-screw fixation), Model **(C)** (L-shaped plate, small fragment, single-screw fixation), and Model **(D)** (T-shaped plate, small fragment, three-screw fixation).

Across all load levels, the displacement distribution patterns were similar for all models, with maximum displacement primarily observed at the tibial plateau load-bearing surface and the fracture fragment region. As the axial load increased from 250 N to 750 N, displacements of the tibia, fracture fragment, and implant progressively increased in each model. Figure 2 illustrates the overall displacement contour plots for the four models under a 750 N load, showing pronounced displacement (red areas) at the tibial plateau, gradually decreasing toward the tibial diaphyseal-metaphyseal junction (blue areas). At 750 N, the overall displacement ranked from highest to lowest as Model B > Model A > Model D > Model C. Figure 3 presents the displacement contour plots for the implants under a 750 N load, with maximum displacement occurring at the proximal end of the plates, corresponding to the region supporting the fracture fragment. Implant displacement at 750 N ranked as Model B > Model A = Model D > Model C. Figure 4 depicts the displacement contour plots for the tibial models under a 750 N load, with displacement concentrated around the fracture fragment fixation site, ranking as Model B > Model A > Model D > Model C.

### Von Mises stress distribution under different loading conditions

**FIGURE 5:**
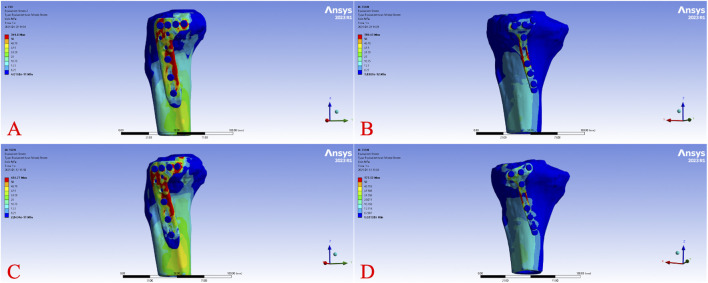
Overall von Mises stress cloud diagram of different fixation models under 750 N axial load. Von Mises stress distribution of the bone-implant construct under a 750 N axial load for Model **(A)** (L-shaped plate, large fragment, dual-screw fixation), Model **(B)** (T-shaped plate, large fragment, four-screw fixation), Model **(C)** (L-shaped plate, small fragment, single-screw fixation), and Model **(D)** (T-shaped plate, small fragment, three-screw fixation).

**FIGURE 6:**
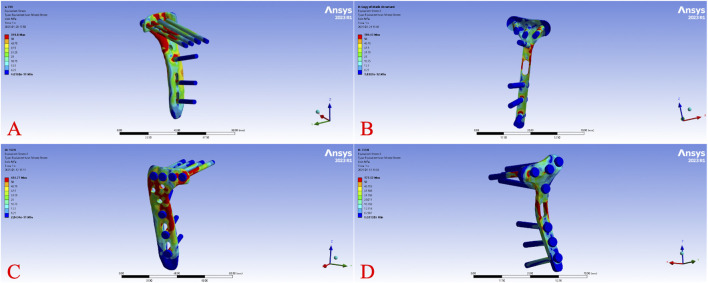
Internal fixation von Mises stress cloud diagram of different fixation models under 750 N axial load. Von Mises stress distribution of the implants (plates and screws) under a 750 N axial load for Model **(A)** (L-shaped plate, large fragment, dual-screw fixation), Model **(B)** (T-shaped plate, large fragment, four-screw fixation), Model **(C)** (L-shaped plate, small fragment, single-screw fixation), and Model **(D)** (T-shaped plate, small fragment, three-screw fixation).

**FIGURE 7:**
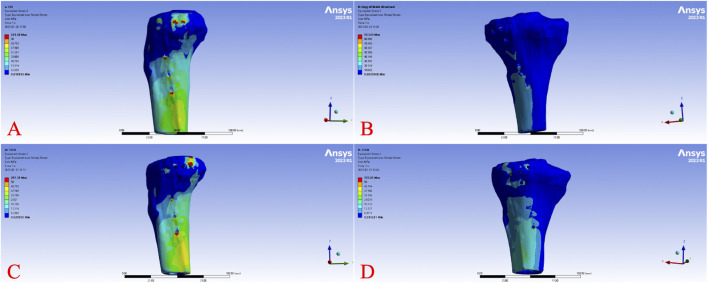
Tibial von Mises stress cloud diagram of different fixation models under 750 N axial load. Von Mises stress distribution of the tibia under a 750 N axial load for Model **(A)** (L-shaped plate, large fragment, dual-screw fixation), Model **(B)** (T-shaped plate, large fragment, four-screw fixation), Model **(C)** (L-shaped plate, small fragment, single-screw fixation), and Model **(D)** (T-shaped plate, small fragment, three-screw fixation).

The von Mises stress distribution across all models under varying loads revealed that stress was primarily concentrated at the screw hole edges of the implants, regions of geometric transition in the plates (e.g., bends), and the screw-bone interface. In the tibial models, stress was predominantly distributed at the load-bearing edges of the fracture fragment and the cortical bone surrounding the screws. Stress values at these sites increased proportionally with higher loads. Figure 5 illustrates the overall von Mises stress contour plots for the four models under a 750 N load. High-stress regions in Model A were concentrated at the plate corner near the last screw and the distal end, while Model B showed stress concentration at the lateral screw holes of the transverse arm and near the plate body. Model C exhibited stress patterns similar to Model A, with additional concentration at the plate body, and Model D displayed stress distribution similar to Model B. At 750 N, the overall von Mises stress ranked from highest to lowest as Model D > Model B > Model A > Model C. Figure 6 presents the von Mises stress contour plots for the implants under a 750 N load. In Model A, high-stress regions were observed at the screw holes of the posterior transverse arm, the midsection of the screws, and the plate corner. Model B showed stress concentration at the lateral screw holes of the transverse arm and the plate body. Model C exhibited stress patterns similar to Model A, but with a notably larger high-stress region at the plate body. Model D displayed stress distribution similar to Model B, with additional high-stress areas at the screw bodies. Figure 7 depicts the von Mises stress contour plots for the tibial models under a 750 N load. In Model A, high-stress regions were observed around the bone near the posterior two screw holes and adjacent to the plate body screw holes. Model C showed stress concentration around the bone near the last screw hole and the plate body screw holes. In contrast, no significant stress concentration was observed in the bone models of Groups B and D.

## Discussion

Posterolateral tibial plateau fractures, due to their anatomical complexity and treatment challenges, remain a focal point in orthopedic research (Sun et al., 2013; Van et al., 2020). Effective internal fixation is critical for restoring joint function and minimizing complications, with fragment size directly influencing the choice of fixation strategy and implant stability (Li et al., 2025; Wang C. S. et al., 2024). This study utilized three-dimensional FEA to compare the biomechanical stability of posterolateral tibial plateau fracture fragments of varying sizes fixed with L-shaped plates or posterior T-shaped tibial plates using different screw configurations. Particular emphasis was placed on the displacement and stress distribution characteristics when reduced fragment size limits the number of screws, aiming to provide a biomechanical basis for clinical treatment decisions.

Under simulated physiological loads (up to 750 N), L-shaped plate fixation demonstrated that, for larger posterolateral fracture fragments allowing dual-screw transverse arm fixation (Model A), implant displacement (0.32 mm) and maximum von Mises stress (349 MPa) were superior to those for smaller fragments limited to single-screw transverse arm fixation (Model C, implant displacement 0.37 mm, maximum stress 454 MPa). However, the displacement difference was minimal, with Model C exhibiting the highest implant stress among all groups. In contrast, posterior T-shaped plate fixation showed comparable performance between larger fragments with four-screw fixation (Model B, implant displacement 0.29 mm, maximum stress 189 MPa) and smaller fragments with three-screw fixation (Model D, implant displacement 0.32 mm, maximum stress 176 MPa), with Model D demonstrating slightly lower implant stress than Model B. Overall, displacement differences across the four models were minimal, and the maximum von Mises stress of all implants under a 750 N load remained well below the yield strength of the titanium alloy used, indicating that all fixation configurations provided acceptable initial biomechanical stability under the tested conditions (Miller and Goswami, 2007).

### Effect of fragment size on L-Shaped plate fixation stability

In this study, when larger posterolateral fracture fragments allowed dual-screw transverse arm fixation with an L-shaped plate (Model A), both maximum implant displacement (0.32 mm) and maximum von Mises stress (349 MPa) demonstrated favorable biomechanical performance. This aligns with established biomechanical principles, where multi-point, distributed screw fixation effectively disperses stress, providing robust stability to maintain fracture fragment alignment and articular surface congruity (Huang et al., 2015; Zeng et al., 2022). However, when fragment size was reduced, limiting the L-shaped plate to single-screw transverse arm fixation (Model C), the maximum implant displacement (0.37 mm) showed only a marginal increase compared to Model A, remaining within a clinically acceptable micro-motion range. In contrast, the maximum von Mises stress in Model C escalated significantly to 454 MPa, the highest among all models, indicating pronounced stress concentration under equivalent loading conditions. Although this stress level remained below the yield strength of the titanium alloy, elevated stress over time may increase the risk of implant fatigue failure or microfractures in the surrounding bone, potentially leading to screw loosening (Wu et al., 2021; Aboushelib and Elraggal, 2023). Clinically, for very small posterolateral fracture fragments accommodating only a single transverse screw with an L-shaped plate, caution is warranted when selecting this fixation method. Considerations should include patient compliance with early weight-bearing restrictions and vigilant monitoring of fracture healing.

### Effect of fragment size on posterior T-Shaped plate fixation stability

In contrast to L-shaped plate fixation, posterior T-shaped plate fixation demonstrated greater adaptability to variations in fragment size in this study. For larger fracture fragments fixed with four proximal screws (Model B), the maximum implant displacement was 0.29 mm, with a maximum von Mises stress of 189 MPa. When fragment size was reduced, limiting fixation to three proximal screws (Model D), the maximum implant displacement slightly increased to 0.32 mm, but the maximum von Mises stress decreased to 176 MPa, with corresponding tibial stress reduced from 190 MPa (Model B) to 104 MPa (Model D). This suggests that, in the design of T-shaped plates, reducing the number of proximal screws from four to three does not significantly compromise overall fixation efficacy, provided the remaining screws effectively engage the fragment and distribute stress. In some cases, this configuration may even yield superior stress distribution by avoiding excessive screw placement in small fragments, which could otherwise lead to stress concentration or additional disruption of the fragment’s blood supply. From the perspective of the “stress shielding effect,” excessive screws may induce abrupt changes in local stiffness, potentially exacerbating stress concentration. The broader proximal design and multiple screw hole rows of posterior T-shaped plates are intended to provide multi-point support and a “raft-like” fixation effect for the posterior plateau (Cho et al., 2017). These findings partially support the notion that T-shaped plates may offer more consistent biomechanical stability and lower implant stress when managing posterolateral fracture fragments of varying sizes, particularly smaller ones.

### Comparative analysis of L-Shaped and T-Shaped plates across different fragment sizes

A comparative analysis of the two plate designs revealed distinct biomechanical performance. For larger fracture fragments (Model A vs. Model B), the posterior T-shaped plate (Model B) exhibited lower maximum implant stress and tibial stress compared to the L-shaped plate (Model A), indicating superior stress distribution characteristics. This advantage was more pronounced for smaller fracture fragments (Model C vs. Model D), where the T-shaped plate (Model D) demonstrated slightly lower maximum implant displacement (0.32 mm vs. 0.37 mm) and substantially lower maximum implant stress (176 MPa vs. 454 MPa) compared to the L-shaped plate (Model C), with tibial stress also significantly reduced. These differences likely stem from the distinct designs of the plates. The L-shaped plate relies heavily on its angular structure and transverse arm screws to resist shear and collapse, with a notable decline in biomechanical performance when the number of transverse screws is reduced (Li et al., 2024). In contrast, the T-shaped plate leverages multiple screws at its plate head to form a cohesive support surface, allowing the remaining screws to maintain structural stability and effectively distribute stress even when one screw is omitted (Lu et al., 2023). These findings suggest that the posterior T-shaped plate may be a more reliable choice for managing posterolateral tibial plateau fractures, particularly when fragment size is small and fixation options are limited.

Our previous studies demonstrated that two-screw fixation provides sufficient mechanical strength for typical posterolateral fracture fragments with adequate volume and mass. The current findings refine this understanding. For larger fragments allowing dual-screw transverse arm fixation with an L-shaped plate (Model A), stability was indeed robust. However, when fragment size was reduced to accommodate only a single transverse screw (Model C), displacement control remained acceptable, but the substantial increase in maximum implant stress suggests potential long-term stability concerns. In contrast, the posterior T-shaped plate exhibited comparable, or even superior, biomechanical performance with smaller fragments fixed using three proximal screws (Model D) compared to four-screw fixation (Model B), particularly in terms of stress distribution. This indicates that the “sufficiency” of screw numbers may depend on a combination of fragment size and plate design, highlighting the need for tailored fixation strategies in clinical practice.

In this study, the maximum implant stress across all models remained below the yield strength of the titanium alloy, indicating that under an instantaneous axial load of 750 N, the implants were unlikely to undergo permanent deformation. However, physiological loading *in vivo* is complex and cyclic, with implant fatigue life closely tied to cyclic stress amplitude (Duan and Griggs, 2018). Model C (L-shaped plate, small fragment) exhibited a peak stress of 454 MPa, which, while safe under single loading, theoretically increases the likelihood of fatigue damage under repetitive loading compared to models with lower stress, such as Model D (T-shaped plate, 176 MPa). Additionally, implant displacements ranging from 0.29 mm to 0.40 mm are generally considered within the acceptable micro-motion range that promotes callus formation at the fracture site, whereas excessive displacement may lead to fixation failure or delayed healing (Rechter et al., 2024). Therefore, evaluating fixation efficacy requires not only assessing immediate mechanical strength but also considering the potential long-term effects of stress levels and displacement magnitude on clinical outcomes.

### Clinical implications and value

The management of posterolateral tibial plateau fractures remains a significant challenge for orthopedic surgeons, particularly when small fracture fragments limit the application of conventional internal fixation methods, necessitating careful selection and placement of implants. This study utilized finite element analysis (FEA) to investigate the influence of fragment size on the biomechanical stability of posterolateral tibial plateau fractures fixed with L-shaped or posterior T-shaped plates, offering valuable insights and practical guidance for optimizing clinical treatment strategies. Firstly, this study provides a biomechanical foundation for selecting internal fixation methods based on fragment size. The results clearly demonstrate that for larger posterolateral fragments accommodating dual-screw transverse arm fixation with an L-shaped plate or four proximal screws with a T-shaped plate, both methods achieve robust initial stability. However, for smaller fragments, which are clinically more challenging, the posterior T-shaped plate with three proximal screws (Model D) exhibited superior stress control and slightly better displacement control compared to the L-shaped plate with a single transverse screw (Model C). This finding suggests that, for small posterolateral fragments with limited bone stock precluding multi-screw L-shaped plate fixation, prioritizing a posterior T-shaped plate with at least three effective screws may represent a safer and more reliable option. This approach could reduce the risk of fixation-related complications, such as loosening or fatigue failure, due to excessive stress concentration. Furthermore, this study highlights potential risks associated with L-shaped plate fixation for very small posterolateral fragments. When limited to a single transverse screw, the L-shaped plate provided acceptable immediate stability (displacement), but the markedly elevated implant stress raises concerns about long-term reliability. This underscores the need for clinicians to thoroughly evaluate patient-specific factors, such as bone quality, anticipated weight-bearing demands, and postoperative rehabilitation protocols, when employing such minimal fixation. Enhanced follow-up is warranted to monitor for early fixation failure in these cases.

### Limitations

This study has several limitations that warrant consideration. First, finite element analysis (FEA) is a theoretical computational method, and the accuracy of its results depends heavily on the precision of model construction and the realism of assigned material properties. Although the tibial models in this study were reconstructed from real computed tomography (CT) data, the bone was assumed to have homogeneous, isotropic, linear elastic properties, which differ from the heterogeneous, anisotropic, and viscoelastic characteristics of human bone. Additionally, fracture lines were simplified as specific geometric gaps, which may not fully replicate the complex morphology and interdigitation of clinical fracture ends. These simplifications may influence the calculated stress and displacement outcomes to some extent. Second, the loading conditions in this study were simplified. The analysis primarily simulated axial compressive loads near the knee’s extended position, a primary load-bearing mode for the tibial plateau. However, it did not fully account for the complex multi-axial loads, shear forces, or dynamic stabilizing effects of muscles and ligaments encountered during actual knee motion (e.g., flexion-extension, rotation) and varying gait cycles. These *in vivo* factors may alter the stress distribution and displacement patterns of the implants and bone. Third, the study utilized specific models of L-shaped and posterior T-shaped plates. Variations in plate design, including material composition, thickness, screw hole arrangement, and pre-contoured angles, across different manufacturers could lead to differing biomechanical performance, limiting the generalizability of our findings. Furthermore, this study focused on immediate biomechanical stability and did not address the dynamic changes during fracture healing or the long-term fatigue performance of the implants. Fracture healing is a complex biological process involving callus formation and bone remodeling, which dynamically alters the mechanical environment of the bone-implant construct. Additionally, patient-specific factors, such as osteoporosis, which may significantly reduce screw purchase and overall fixation stability, were not considered. Finally, the definitions of “large” and “small” fracture fragments in this study were relative, based on the ability to accommodate specific screw numbers. In clinical practice, fracture fragment size and morphology are highly variable, and the two models evaluated here may not encompass the full spectrum of clinical scenarios.

## Conclusion

This study demonstrates that fragment size significantly influences the biomechanical stability of different internal fixation methods for posterolateral tibial plateau fractures. For larger fragments, both L-shaped plates with dual-screw transverse arm fixation and posterior T-shaped plates with four proximal screws provided reliable initial stability. However, when fragment size is reduced, limiting L-shaped plates to single-screw transverse arm fixation, implant stress markedly increases, suggesting a potential risk of fatigue failure. In contrast, posterior T-shaped plates maintained favorable biomechanical stability even with smaller fragments fixed using three proximal screws, exhibiting lower implant stress levels. Therefore, for clinical management of posterolateral tibial plateau fractures, particularly when small fragment size restricts fixation options, posterior T-shaped plates, even with a reduced number of proximal screws, may represent a biomechanically superior choice. These findings offer valuable guidance for individualized treatment strategies, although further clinical studies are needed to validate these results.

## Data Availability

The original contributions presented in the study are included in the article/Supplementary Material, further inquiries can be directed to the corresponding author.
