# Supplementary material for: Finite element analysis of the influence of fragment size on biomechanical outcomes of various fixation techniques for posterolateral tibial plateau fractures
Source: Front Bioeng Biotechnol. 2026 Jan 28;14:1676886. doi: 10.3389/fbioe.2026.1676886 (PMC12891237; doi:10.3389/fbioe.2026.1676886)
Supplement: Supplementary file 1 [file Table1.docx]

| Parameter | Lateral L-shaped locking plate | Posterior T-shaped locking plate |
| --- | --- | --- |
| Manufacturer | Kanghui Medical (China) | Kanghui Medical (China) |
| Product model | Lateral tibial plateau L-shaped locking compression plate (left/right configuration; multiple hole numbers and plate lengths available; compatible with locking screws) | Posterior tibial plateau T-shaped locking compression plate (left/right configuration; multiple hole numbers and plate lengths available; compatible with locking screws) |
| Regulatory approval | National Medical Products Administration (NMPA) registration No. 20183131799 | National Medical Products Administration (NMPA) registration No. 20183131799 |
| Structural design | Composed of a locking plate and locking screws; angular stability achieved through the engagement between the screw head threads and plate holes; anatomically contoured plate body with guiding/combination holes, allowing use with a targeting device | Composed of a locking plate and locking screws; angular stability achieved through internal–external thread locking; anatomically contoured T-shaped plate body providing multiple proximal screw options and compatibility with a targeting device |
| Material | Medical-grade commercially pure titanium (conforming to GB/T 13810) | Medical-grade commercially pure titanium (conforming to GB/T 13810) |
| Indications | Intended for internal fixation of limb fractures (used in this study for posterolateral tibial plateau fractures) | Intended for internal fixation of limb fractures (used in this study for posterolateral tibial plateau fractures) |
| Biocompatibility | Evaluated according to GB/T 16886 / ISO 10993 standards; meets requirements for implantable medical devices | Evaluated according to GB/T 16886 / ISO 10993 standards; meets requirements for implantable medical devices |
| Labeled adverse reactions | Potential risks include infection, hypersensitivity or metal allergy, pain or swelling, local irritation, nonunion, implant loosening, or implant breakage | Potential risks include infection, hypersensitivity or metal allergy, pain or swelling, local irritation, nonunion, implant loosening, or implant breakage |

Table 1. Material and structural characteristics of the implants used in this study
